# Chasing the spin gap through the phase diagram of a frustrated Mott insulator

**DOI:** 10.1038/s41467-023-37491-z

**Published:** 2023-04-07

**Authors:** A. Pustogow, Y. Kawasugi, H. Sakurakoji, N. Tajima

**Affiliations:** 1 Institute of Solid State Physics, TU Wien, 1040 Vienna, Austria; 2 Department of Physics, Toho University, Funabashi, 274-8510 Chiba Japan; 3 Condensed Molecular Materials Laboratory, RIKEN, Wako, Saitama 351-0198 Japan

**Keywords:** Magnetic properties and materials, Electronic properties and materials, Phase transitions and critical phenomena, Quantum fluids and solids

## Abstract

The quest for entangled spin excitations has stimulated intense research on frustrated magnetic systems. For almost two decades, the triangular-lattice Mott insulator *κ*-(BEDT-TTF)_2_Cu_2_(CN)_3_ has been one of the hottest candidates for a *gapless* quantum spin liquid with itinerant spinons. Very recently, however, this scenario was overturned as electron-spin-resonance (ESR) studies unveiled a spin gap, calling for reevaluation of the magnetic ground state. Here we achieve a precise mapping of this spin-gapped phase through the Mott transition by ultrahigh-resolution strain tuning. Our transport experiments reveal a reentrance of charge localization below *T*^⋆^ = 6 K associated with a gap size of 30–50 K. The negative slope of the insulator-metal boundary, *dT*^⋆^/*dp* < 0, evidences the low-entropy nature of the spin-singlet ground state. By tuning the enigmatic ‘6K anomaly’ through the phase diagram of *κ*-(BEDT-TTF)_2_Cu_2_(CN)_3_, we identify it as the transition to a valence-bond-solid phase, in agreement with previous thermal expansion and magnetic resonance studies. This spin-gapped insulating state persists at *T* → 0 until unconventional superconductivity and metallic transport proliferate.

## Abstract (web summary)

The Mott insulator *κ*-(BEDT-TTF)_2_Cu_2_(CN)_3_ has been a strong candidate for a gapless quantum spin liquid, but recent experiments suggested a spin-gapped phase below 6 K. Pustogow et al. study the entropy of this phase by driving the system through the metal-insulator transition with a strain engineering approach.

## Introduction

Since Anderson’s notion of resonating valence bonds^1^, the quest for quantum spin liquids (QSL) has been fueled by the idea that suppressing long-range antiferromagnetic (AFM) order can stabilize itinerant or even topological spin excitations^2,3^. What is often neglected is that the coupling of magnetic degrees of freedom to the lattice can result in valence-bond-solid (VBS) phases with paired electron spins. Such singlet states occur, for instance, in triangular-lattice organic compounds^4–8^ and in form of the well-known spin-Peierls (SP) states in quasi 1D systems^9,10^. Also in other frustrated materials the importance of valence-bond phases has been revived^11–15^. Such a VBS scenario is fully consistent with the report of a spin gap in the organic QSL candidate *κ*-(BEDT-TTF)_2_Cu_2_(CN)_3_ that aroused great attention in the QSL community very recently^16,17^, in agreement with gapped excitations in thermal transport^18^ and a phase transition at 6 K deduced from thermal expansion^19^ and NQR measurements^20^. While this finding rules out a gapless scenario with mobile spinons, that would have explained the finite Sommerfeld coefficient in specific heat results^21^, it leaves open the possibility of gapped QSL phases^3,22–24^.

**Fig. 1:**
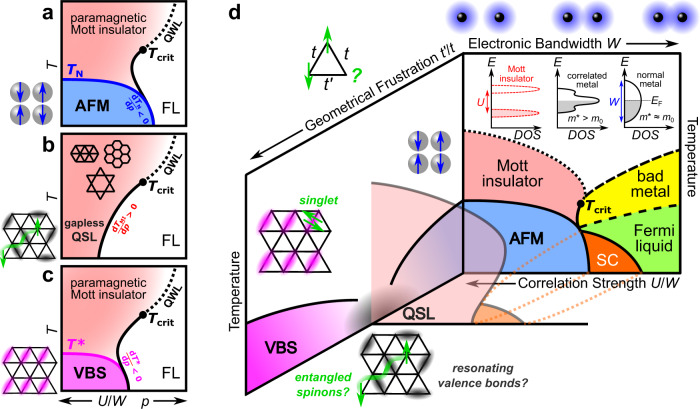
Phase diagram of Mott insulators as function of frustration and correlation strength. **a** While the entropy of a paramagnetic Mott insulator exceeds that of the adjacent Fermi liquid (FL), resulting in a positive slope *dT*_MI_/*dp* > 0 of the metal-insulator boundary, AFM order has much smaller entropy and the Clausius-Clapeyron relation yields *dT*_N_/*dp* < 0, as seen in *κ*-(BEDT-TTF)_2_Cu[N(CN)_2_]Cl^36,37^. **b** No such 'reentrance' of insulating behavior is expected for a *gapless* QSL, possibly realized in triangular, kagome or honeycomb lattices^2,3^. **c** Similar to AFM, also the transition from a spin-gapped VBS insulator to a metal yields *dT*^⋆^/*dp* < 0, for instance in EtMe_3_P[Pd(dmit)_2_]_2_^5,6^. **d** Geometrical frustration ${t}^{{\prime} }/t$ controls the magnetic ground state of triangular-lattice Mott insulators, causing pronounced changes in the phase diagram affecting also unconventional superconductivity (SC). Experimentally, AFM has been observed for ${t}^{{\prime} }/t < 1$ in *κ*-(BEDT-TTF)_2_Cu[N(CN)_2_]Cl and ${\beta }^{{\prime} }$-[Pd(dmit)_2_]_2_ salts whereas magnetic order is absent in the QSL candidates *κ*-(BEDT-TTF)_2_Cu_2_(CN)_3_, *κ*-(BEDT-TTF)_2_Ag_2_(CN)_3_ and EtMe_3_Sb[Pd(dmit)_2_]_2_ with ${t}^{{\prime} }/t$ close to unity^26,38^; VBS states were reported for EtMe_3_P[Pd(dmit)_2_]_2_ (${t}^{{\prime} }/t\approx 1$)^4^ and *κ*-(BEDT-TTF)_2_B(CN)_4_ (${t}^{{\prime} }/t\approx 1.4$)^8^. The frustration dependence of AFM and VBS phases is schematically indicated for these systems, calling for in-depth studies upon controlled variation of ${t}^{{\prime} }/t$.

The tunability of *κ*-(BEDT-TTF)_2_Cu_2_(CN)_3_—a pressure of 1.3 kbar triggers the transition from a Mott insulator to superconducting (SC) and metallic phases^5,6,25–28^ as illustrated in Fig. 1d—provides the unique opportunity to scrutinize the entropy of its magnetic ground state. According to the Clausius-Clapeyron relation, the pressure-tuned metal-insulator transition (MIT) acquires a positive slope *dT*_MI_/*dp* > 0 in the *T*-*p* phase diagram between a paramagnetic Mott insulator with large spin entropy and a metal (Fig. 1b), reminiscent of the Pomeranchuk effect in ^3^He^28^. On the other hand, the entropy of AFM and VBS states is smaller than that of the Fermi liquid (FL) metal, yielding negative slopes *dT*_N_/*dp* < 0 and *dT*^⋆^/*dp* < 0 of the MIT (Fig. 1a, c), respectively.

Here we utilize the slope of the insulator-metal boundary of *κ*-(BEDT-TTF)_2_Cu_2_(CN)_3_ as a probe of the spin-gapped ground state below *T*^⋆^ = 6 K, evidencing its low-entropy nature. For that, we map the low-temperature Mott MIT with ultrahigh precision by measuring dc transport upon a combination of biaxial compression and tensile strain. We find a reentrance of insulating behavior setting in below *T*^⋆^, which is successively suppressed as metallicity proliferates. Through comparison with previously reported structural^7,19^ and magnetic^16^ properties, our present results clearly identify the ’6K anomaly’ as the transition to a VBS phase with *dT*^⋆^/*dp* < 0 (Fig. 1c) and, thus, we complete the phase diagram of *κ*-(BEDT-TTF)_2_Cu_2_(CN)_3_. Moreover, our transport data yield a gap size of 30–50 K for the spin-singlet state, which coincides with the unexpectedly large critical field of 60 T^17^.

## Results

Among the QSL candidates, *κ*-(BEDT-TTF)_2_Cu_2_(CN)_3_ has been studied most intensely as it exhibits not only frustrated AFM exchange interactions (*J* = 250 K^29^), but also a paradigmatic Mott transition^25,28,30^. This layered triangular-lattice compound with ${t}^{{\prime} }/t$ close to unity^31,32^ shows no AFM order^29^ and, at the same time, it is one of the best solid-state realizations of the single-band Hubbard model^28^. As such, it provides experimental access to check the predictions of dynamical mean-field theory: while the Mott transition is first-order type below the critical endpoint *T*_*crit*_, a quantum-critical crossover at the quantum Widom line (QWL) occurs at *T* > *T*_*crit*_^28,30,33,34^. Moreover, applying 1–2 kbar pressure allows to probe unconventional SC^25,27^ as well as charge transport in Fermi liquids and *bad* metals^35^. By combining the most recent findings in *κ*-(BEDT-TTF)_2_Cu_2_(CN)_3_^28,30,35^ and compounds with different degree of frustration^5,8,26,30,36–38^, in Fig. 1d we present the state-of-the-art phase diagram of dimerized organic Mott systems as a function of *T*, electronic correlations *U*/*W* and geometrical frustration ${t}^{{\prime} }/t$.

**Fig. 2:**
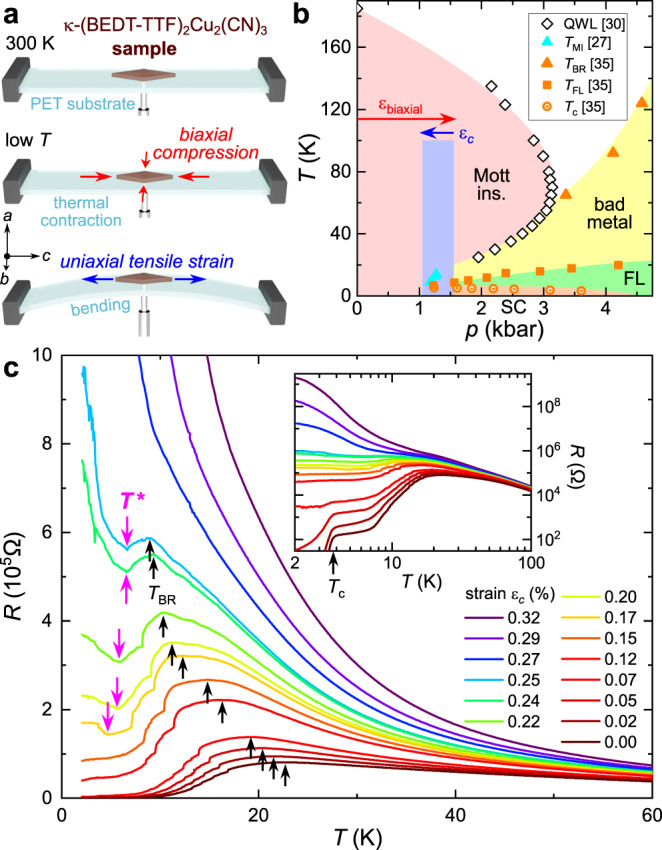
Strain-tuning of *κ*-(BEDT-TTF)_2_Cu_2_(CN)_3_. **a**, **b** A 140 nm thin single-crystalline filament of *κ*-(BEDT-TTF)_2_Cu_2_(CN)_3_ was placed on a flexible PET substrate. Cooling from 300 K down to *T* → 0 imposes biaxial compression of order 1–2 kbar to the sample due to differential thermal contraction, yielding metallic and superconducting (SC) properties (red arrow in **b**). Uniaxial tensile strain *ε*_*c*_ along the *c*-axis is applied via bending of the substrate in order to tune from the Fermi liquid (FL) through the Mott MIT (blue arrow in **b**). **b** Crossover and transition temperatures from refs. ^27,30,35^, as indicated. **c** Resistance of the biaxially compressed crystal for different *ε*_*c*_. Inset: SC below *T*_*c*_ ≈ 4 K for *ε*_*c*_ = 0 is evident in the log-log plot. Upon approaching the Mott state, SC is lost and the resistivity maxima at *T*_BR_ shift to lower temperatures. In addition, our data reveal a reentrance of insulating behavior (magenta arrows) below *T*^⋆^ < *T*_BR_.

In this study, we explore the phase space extremely close to the MIT, which requires fine tuning of the correlation strength with a resolution equivalent to 10^−2^ kbar. While such high precision cannot be reached in conventional oil pressure cells^25,35^, also gas pressure experiments are limited at low temperatures due to solidification of helium^27,30^. To that end, we utilize strain transmitted through a substrate—a method previously applied in doping-tuned experiments^39^. As sketched in Fig. 2a, a single-crystalline *κ*-(BEDT-TTF)_2_Cu_2_(CN)_3_ filament of 140 nm thickness is placed on a flexible polyethylene substrate at ambient conditions. Differential thermal expansion between sample and substrate (see Methods) causes an in-plane biaxial compression *ε*_biaxial_ equivalent to 1–2 kbar hydrostatic pressure, which is sufficient to push the sample across the Mott MIT (Fig. 2b). Our dc transport results in Fig. 2c yield metallic properties and an onset of SC below *T*_*c*_ ≈ 4 K (*ε*_*c*_ = 0, see inset). Note, uniaxial strain experiments on *κ*-(BEDT-TTF)_2_Cu_2_(CN)_3_ revealed a considerable enhancement of *T*_*c*_ and a broadened SC dome^40^ than for isotropic compression. Here, biaxial strain does not affect the in-plane anisotropy and *T*_*c*_ is comparable to hydrostatic pressure experiments^25,27^.

While differential thermal contraction tunes the sample across the Mott MIT, it does not allow to vary the correlation strength in arbitrary steps. In order to reach back into the Mott state in a quasi-continuous manner, we perform bandwidth-tuning via tensile strain *ε*_*c*_ > 0 along the crystallographic *c*-axis. As indicated on the bottom of Fig. 2a, we control the correlation strength by varying the bending radius of the substrate, which yields a *negative* pressure of less than a kilobar. As such, uniaxial strain is significantly weaker than the overall pressure applied (∣*ε*_*c*_∣ < ∣*ε*_biaxial_ + *ε*_*c*_∣), hence we do not expect major effects from a change in frustration ${t}^{{\prime} }/t$—certainly less pronounced than in ref. ^40^. The blue shaded region in Fig. 2b indicates the phase space covered in our experiments with a fixed *ε*_biaxial_ < 0 and varying tensile strain between *ε*_*c*_ = 0 and 0.32%. The resulting transport curves in Fig. 2c exhibit a textbook Mott MIT: the resistivity maxima at the Brinkman-Rice temperature *T*_BR_ are pushed to lower temperatures as correlations increase^25,35^. In addition, in the range between *ε*_*c*_ = 0.17% and 0.25% we observe a distinct upturn of resistance upon cooling well below *T*_BR_. The resistance minima (magenta arrows in Fig. 2c) shift up to *T*^⋆^ = 6 K until metallic behavior is lost completely. Since this phenomenology lines up with the ‘6K anomaly’ at ambient pressure, we identify it as the transition to a spin-gapped non-metallic state that gets suppressed at the Mott MIT. Note, a similar upturn of resistivity around the MIT has been recently observed in a pressure-dependent transport study on the sister compound *κ*-(BEDT-TTF)_2_Ag_2_(CN)_3_^41^, which calls for further scrutiny.

**Fig. 3:**
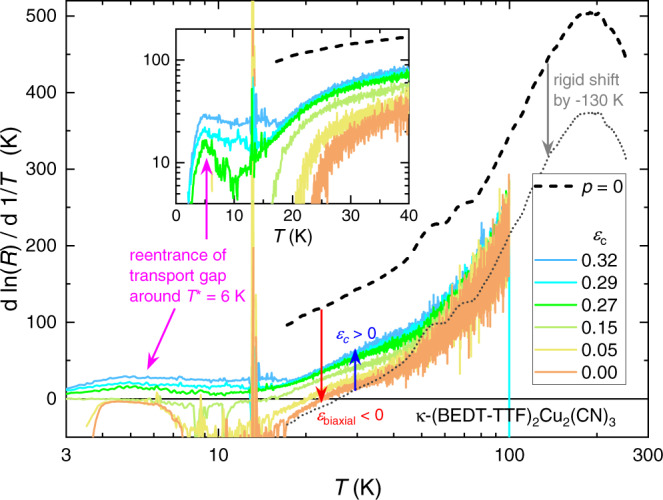
Fingerprints of Mott transition and ‘6K anomaly’ in transport gap. The transport gap $d\ln R/d\ 1/T$ determined from the strain-dependent resistance (Fig. 2c) is compared to ambient pressure (black dashed line from ref. ^28^). While biaxial compression *ε*_biaxial_ < 0 causes a reduction of the gap size, equivalent to a vertical shift of the *p* = 0 data by −130 K (gray dotted), the gap increases upon tensile strain *ε*_*c*_ > 0, consistent with the bandwidth-tuning in Fig. 2b. The Mott MIT is evident from the sign change at *T*_BR_. Upon further cooling in the metallic state, $d\ln R/d\ 1/T\to 0$ as the residual resistivity is approached, followed by a sharp drop when SC sets in. Notably, the fully insulating curves exhibit a local maximum around *T*^⋆^ = 6 K associated with the VBS transition.

The reentrant behavior at low temperatures is apparent not only from a sign change of *dR*/*dT* to a non-metallic slope (*dR*/*dT* < 0 below *T*^⋆^), but can be seen also in the fully insulating curves *ε*_*c*_ ≥ 0.27%. In Fig. 3 we plot the logarithmic derivative $d\ln R/d\ 1/T$ as a measure of the transport gap Δ(*T*). While the gap size is steadily reduced upon cooling from 100 K to 20 K, consistent with the temperature-dependent spectral weight shifts reported in previous optical studies^28,35^, we observe an upturn at low temperatures forming a peak around *T*^⋆^. For comparison, the VBS transition of EtMe_3_P[Pd(dmit)_2_]_2_ at *T*^⋆^ = 24 K yields similar maxima in $d\ln R/d\ 1/T$ from which we estimate a ratio of gap size and transition temperature Δ_*VBS*_/(*k*_*B*_*T*^⋆^) ≈ 5^5^. Assuming the same ratio in the title compound gives Δ_*VBS*_/*k*_*B*_ ≈ 30 K for *T*^⋆^ = 6 K, which compares well with the local maxima in the inset of Fig. 3.

In ambient pressure transport studies^28^, the regime *T* < 10 K remained inaccessible due to the extremely large resistance; here, in the vicinity of the MIT *R*(*T*) is small enough to be measured. Following its observation in thermodynamic and magnetic probes^16,19,21^, in our present work we identify the ‘6K anomaly’ in the Mott-insulating state of *κ*-(BEDT-TTF)_2_Cu_2_(CN)_3_ for the first time in charge transport as a local maximum of $d\ln R/d\ 1/T$. Even more importantly, we obtain a quantitative estimate of the associated energy gap *E*_*g*_ = 2Δ ≈ 30–50 K at *T*^⋆^. Note, this significantly exceeds the spin gap of 12 K estimated from ESR data^16^, which may indicate the involvement of other degrees of freedom (e.g., structural) in the transition in addition to magnetic interactions. Crucially, the gap size observed here lines up with the critical field of order 60 T estimated in ref. ^17^, which explains why magnetic fields of order 10 T have little effect on the transition^19,21^.

$d\ln R/d\ 1/T$ in the range *T* > 20 K reflects changes of the Mott-Hubbard gap. While the gap size reduces upon biaxial compression, it increases upon uniaxial tension; the negative values below *T*_BR_ correspond to *dR*/*dT* > 0. Interestingly, the strain-induced change upon *ε*_biaxial_ < 0 is approximately a rigid vertical shift of the ambient pressure data^28^ by −130 K (gray dotted line in Fig. 3). Quantitatively, the effect of *ε*_biaxial_ is approximately three times bigger than *ε*_*c*_ = 0.32%, consistent with the red and blue arrows in Fig. 2b, respectively.

**Fig. 4:**
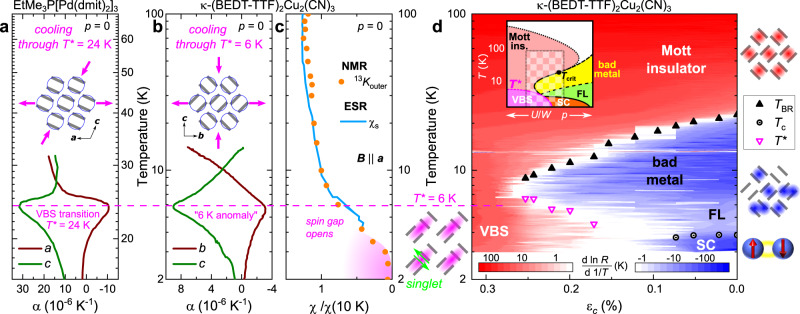
Transition to valence-bond-solid (VBS) state at ‘6K anomaly’. **a**, **b** The thermal expansion coefficient *α* yields similar in-plane anisotropy at the VBS transition of EtMe_3_P[Pd(dmit)_2_]_2_ (*T*^⋆^ = 24 K)^7^ and at the ‘6K anomaly’ of *κ*-(BEDT-TTF)_2_Cu_2_(CN)_3_^19^. Note the different scales; data from refs. ^7,19^. **c** The rapid drop of NMR Knight shift *K* and ESR susceptibility *χ*_*s*_ (*B*∥*a*) at *T*^⋆^ = 6 K evidences the opening of a spin gap; data from refs. ^16,44^. **d** The phase diagram of *κ*-(BEDT-TTF)_2_Cu_2_(CN)_3_ assembled from *R*(*T*, *ε*_*c*_) (Figs. 2c and 3) is underlaid with a false-color plot of $d \, {{{{{\rm{ln}}}}}} \, R/d \, 1/T$ (red: insulating, blue: metallic). Inset: checkerboard area indicates range of our strain-dependent experiments. We find a clear back-bending of the insulator-metal boundary (cf. Fig. 1c) originating from the ‘6K anomaly’ at *p* = 0. Altogether, the ambient pressure data in **b**, **c** and *dT*^⋆^/*dp* < 0 in **d** provide solid evidence for a low-entropy spin-singlet phase with structural anisotropy -- the hallmarks of a VBS.

In Fig. 4a, b we compare the structural changes at the ‘6K anomaly’ in *κ*-(BEDT-TTF)_2_Cu_2_(CN)_3_ with the VBS transition at *T*^⋆^ = 24 K in EtMe_3_P[Pd(dmit)_2_]_2_^4^. Thermal expansion experiments by Manna et al.^7,19^ revealed very similar anisotropic distortions within the conducting layers at *T*^⋆^ in both quasi 2D organic compounds. As sketched in Fig. 4a, b, the crystal contracts along the *c*-axis and expands along the other in-plane direction upon cooling. Also other probes^20,42,43^ observed pronounced anomalies at 6 K due to modifications of the crystal structure and possible symmetry breaking^17^, which are typical fingerprints of a VBS transition. In addition, recent ESR studies revealed a rapid drop of spin susceptibility *χ*_*s*_ due to the opening of a spin gap^16^, which agrees with NMR Knight shift data from ref. ^44^ that yield *χ*_*s*_ indistinguishable from zero for *T* → 0^17^, as shown in Fig. 4c.

Altogether, these findings provide solid evidence for anisotropic structural changes at the transition to a singlet state with a gap in the spectrum of spin excitations. In addition, *T*^⋆^ = 6 K at *p* = 0 directly interpolates to the reentrance of the MIT in our strain-dependent transport data, which is mapped in the false-color plot of $d\ln R/d\ 1/T$ in Fig. 4d. We find that the resulting phase diagram prominently displays *dT*^⋆^/*dp* < 0 as sketched in Fig. 1c, analog to the VBS Mott insulator EtMe_3_P[Pd(dmit)_2_]_2_^5,6^ with similar thermal expansion anomalies (Fig. 4a, b). In light of these overwhelming experimental similarities, we conclude that at ambient pressure the enigmatic ‘6K anomaly’ in *κ*-(BEDT-TTF)_2_Cu_2_(CN)_3_ is the transition from a paramagnetic Mott insulator to a spin-gapped VBS state. Upon reducing the correlation strength, the VBS phase is suppressed and can be entered from a metallic state upon cooling, as observed here for 0.17% ≤ *ε*_*c*_ ≤ 0.25%. As seen in Fig. 4d, in the limit *T* → 0 the nonmagnetic insulator is adjacent to the Cooper-paired state below *T*_*c*_ ≈ 4 K, hence the ’6K anomaly’ may affect unconventional SC.

## Discussion

Since the first reports of the ‘6K anomaly’ and the absence of magnetic order^21,29^, there have been different interpretations of the experimental results acquired over two decades in independent groups^16–19,44^. Here, we put together now all these pieces and obtain a comprehensive understanding of the low-temperature physics of *κ*-(BEDT-TTF)_2_Cu_2_(CN)_3_. Our present ultrahigh-resolution tuning of the ’6K anomaly’ through the Mott MIT delivers the missing part required to establish it as a true phase transition to a low-entropy spin-singlet ground state with distorted crystal structure. In view of the very shape of the phase diagram presented here, the magnetic, thermal transport, thermodynamic and structural properties of *κ*-(BEDT-TTF)_2_Cu_2_(CN)_3_^16,18–20,29,43,44^ are consistent with a spin-gapped VBS state, essentially identical to that reported in other layered organic Mott systems^4–8^. While chiral QSL states with a spin gap^22–24^ cannot be ruled out at present, it is unclear whether the distinct lattice effects of the ‘6K anomaly’^19^ are compatible with these theoretical models. In absence of a ’smoking-gun’ experiment deciding between the two possibilities, the principle of *Occam’s Razor*^45^ suggests to favor not the exotic (chiral QSL) but rather the established scenario (VBS), not least as the latter has already been observed in related triangular-lattice systems^4–8^.

As such, we point out that the spin-gapped low-entropy phase associated with the ’6K anomaly’ is not intrinsic to *pure* Mott insulators, but rather results from magneto-structural instabilities. Likely, the elastic energy involved in the lattice distortion at *T*^⋆^ contributes to the unexpectedly large energy gap and critical field, exceeding the spin gap deduced from ESR data^16^ and *k*_*B*_*T*^⋆^/*μ*_*B*_ ≈ 9 T by far^17^. Previous works have suggested the involvement of charge degrees of freedom^19,20,43^, but a sizeable charge disproportionation has been ruled out by vibrational spectroscopy^46^. The recent observation of reentrant insulating behavior in the related QSL candidate *κ*-(BEDT-TTF)_2_Ag_2_(CN)_3_ possibly provides another test bed to investigate these issues^41^.

Finally, we suggest that, in absence of the VBS, the metallic state would persist much deeper into the insulating region of the phase diagram—in Fig. 2b *T*_FL_ and *T*_BR_ extrapolate to zero well below *p* = 1 kbar^27,35^. To that end, elucidating the *genuine* Mott MIT in the limit *T* → 0 requires to suppress *T*^⋆^ in *κ*-(BEDT-TTF)_2_Cu_2_(CN)_3_, potentially by varying ${t}^{{\prime} }/t$ through uniaxial strain (Fig. 1d) or by applying high magnetic fields *B* ≈ 50–100 T. To conclude, our findings highlight the importance to gain understanding and control of magneto-elastic coupling in frustrated spin systems, in particular in other QSL candidates.

## Methods

### Sample preparation and application of uniaxial bending strain

We prepared polyethylene terephthalate (PET) substrates (Teflex® FT7, Toyobo Film Solutions Limited) and patterned 18-nm-thick Au electrodes using photo-lithography. Thin single crystals of *κ*-(BEDT-TTF)_2_Cu_2_(CN)_3_ were synthesized with electrolysis of a 1,1,2-trichloroethane [10% (v/v) ethanol] solution (50 ml), in which BEDT-TTF (20 mg), KCN (60mg), CuCN (40 mg), and 18-crown-6 (100 mg) are dissolved. We applied a current of 8 μA overnight and obtained tiny thin crystals of *κ*-(BEDT-TTF)_2_Cu_2_(CN)_3_. The crystals were pipetted to 2-propanol, and were manipulated with a tip of hair and put on the substrate. After the substrate was taken out from 2-propanol and dried, the thin crystal tightly adhered to the substrate.

As described in previous work^39,47,48^, cooling yields differential thermal expansion between sample and substrate: the length change is ≤1% between 0 K and 300 K for *κ*-(BEDT-TTF)_2_Cu_2_(CN)_3_^19,49^ compared to >>1% for PET. As a result, the sample is subject to biaxial compression of order 1–2 kbar, deduced from the transport results in Fig. 2 in comparison to previously published resistivity data^27,30,35^. We applied uniaxial bending strains by pushing the back of the substrate with a nanopositioner (ANPz51, attocube systems) as shown in Fig. 2a. Assuming that the bent substrate is an arc of a circle, the strain *S* is estimated as *S* = 4*tx*/(*l*^2^ + 4*x*^2^)^47,48^ using the small angle approximation, where *x* is the displacement of the nanopositioner, *t* = 177 μm and *l* = 12 mm are the thickness and length of the substrate, respectively. The uniaxial tensile strain was applied at 100 K in descending order from 0% to 0.32%. The cooling rate of the sample was 0.5 K/min. The thickness of the studied *κ*-(BEDT-TTF)_2_Cu_2_(CN)_3_ crystal (140 nm) was measured *a posteriori* using a step profiler. Throughout the manuscript we follow the convention that a reduction of the sample volume (upon compressive stress or pressure) corresponds to negative strain *ε* < 0, whereas an expansion of the sample size (upon tensile stress or ‘negative’ pressure) yields *ε* > 0. By comparing our results to transport measurements under hydrostatic pressure^30,35^, we estimate that the total applied strain *ε*_*biaxial*_ + *ε*_*c*_ corresponds to a hydrostatic pressure *p* = 1.55 kbar for *ε*_*c*_ = 0, and *p* = 1.05 kbar for *ε*_*c*_ = 0.32%, yielding a conversion factor Δ*ε*_*c*_/Δ*p* ≈ − 0.6 %/kbar. The range (1.05–1.55 kbar) covered by tuning of *ε*_*c*_ in Figs. 2c and 4d is indicated by the blue rectangle in Fig. 2b.

### Data analysis

The QWL in Fig. 2b was taken from ref. ^28^ whereas *T*_BR_, *T*_*c*_, and *T*_FL_ are from ref. ^35^ and the insulator-metal boundary below 20 K (cyan) was determined from the resistivity data in ref. ^27^. The transport gap of *κ*-(BEDT-TTF)_2_Cu_2_(CN)_3_ at ambient pressure in Fig. 3 (dashed black line) was taken from Fig. S2 in the Supplementary Information of ref. ^28^; the gray dotted line is a rigid vertical shift of the ambient pressure data by −130 K: Δ(*ε*_biaxial_, *T*) ≈ Δ(*p* = 0, *T*) − 130 K. Thermal expansion data in Fig. 4a are from ref. ^7^ and the data in panel b are from ref. ^19^. The NMR shifts in Fig. 4c were taken from ref. ^44^ and the Knight shift *K*_*outer*_ was determined for the outer ^13^C nuclei using a chemical shift σ = 117, as described in ref. ^17^. In the same panel, the ESR susceptibility *χ*_*s*_ for magnetic field perpendicular to the layers (*B*∥*a* similar to the Knight shift data) was taken from Fig. 2a in ref. ^16^. Both *χ*_*s*_ and *K*_*outer*_ were normalized to their value at 10 K.

At elevated temperatures the resistivity maxima indicate *T*_BR_^35^. At lower temperatures in the phase coexistence regime *T* ≤ *T*_*crit*_ ≈ 15 K^50^, strictly speaking, the resistivity maxima indicate percolation of metallic regions. We assign the sharp jumps in the resistance data in Fig. 2c, also seen as narrow peaks between 10–15 K in Fig. 3, to an artefact of the sample mounting. As such, the apparent strain dependence is naturally related to the volume change of the sample that interferes with the applied strain. A micro-crack will open or close for a particular differential thermal expansion between sample and substrate, reached at a different temperature for different strain conditions upon changing *ε*_*c*_.

## Data Availability

The authors declare that the data supporting the findings of this study are available within the paper. Further information can be provided by A.P. and Y.K. upon request.
